# Neutrophils in chronic inflammatory diseases

**DOI:** 10.1038/s41423-021-00832-3

**Published:** 2022-01-17

**Authors:** Andrea Herrero-Cervera, Oliver Soehnlein, Ellinor Kenne

**Affiliations:** 1 Institute for Experimental Pathology, Center for Molecular Biology of Inflammation, Westfälische Wilhelms-Universität Münster, Münster, Germany; 2 Department of Physiology and Pharmacology, Karolinska Institutet, Stockholm, Sweden

**Keywords:** Chronic, Inflammation, Disease, Neutrophil, Neutrophil extracellular traps, Chronic inflammation, Mechanisms of disease

## Abstract

Chronic inflammation is a component of many disease conditions that affect a large group of individuals worldwide. Chronic inflammation is characterized by persistent, low-grade inflammation and is increased in the aging population. Neutrophils are normally the first responders to acute inflammation and contribute to the resolution of inflammation. However, in chronic inflammation, the role of neutrophils is less well understood and has been described as either beneficial or detrimental, causing tissue damage and enhancing the immune response. Emerging evidence suggests that neutrophils are important players in several chronic diseases, such as atherosclerosis, diabetes mellitus, nonalcoholic fatty liver disease and autoimmune disorders. This review will highlight the interaction of neutrophils with other cells in the context of chronic inflammation, the contribution of neutrophils to selected chronic inflammatory diseases, and possible future therapeutic strategies.

## Introduction

The acute inflammatory process is a vital response to injury and infection that protects the organism against invading pathogens and repairs tissue damaged by trauma. Acute inflammation is self-limiting with a resolution phase that terminates the inflammatory response and initiates the reparative process [1, 2]. Chronic inflammation occurs if the initiating stimulus is not removed or if the resolution program is disturbed, resulting in a state of low-grade inflammation. Chronic inflammatory diseases, including atherosclerosis, diabetes mellitus, nonalcoholic fatty liver disease (NAFLD) and autoimmune disorders, are major causes of death worldwide [3]. Systemic chronic inflammation increases with age [4], is low-grade and persistent, and the causes include chronic infections, lifestyle and environmental factors, physical inactivity, microbiome dysbiosis, diet, psychological stress, and toxins [5].

Neutrophils, which are the most abundant white blood cells in humans, have been well established as first responders to acute inflammation. Under normal conditions, neutrophils contribute to resolution and tissue repair by phagocytosing necrotic cells to stop them from attracting more immune cells, releasing mediators to promote growth and angiogenesis, and producing resolvins and protectins [6]. Over the past decades, neutrophils have also been shown to play a significant role in chronic inflammation. Neutrophils are continuously recruited to the site of chronic inflammation and contribute to driving the process through the release of serine proteases and the formation of neutrophil extracellular traps (NETs), as well as the activation of other immune cells. Here, we will review neutrophil crosstalk with other cells in the context of chronic inflammation and the contribution of neutrophils to selected chronic inflammatory diseases and summarize potential strategies to interfere with the damaging effects of neutrophils in chronic diseases.

## Neutrophil networks

### Neutrophil-platelet interactions

**Fig. 1:**
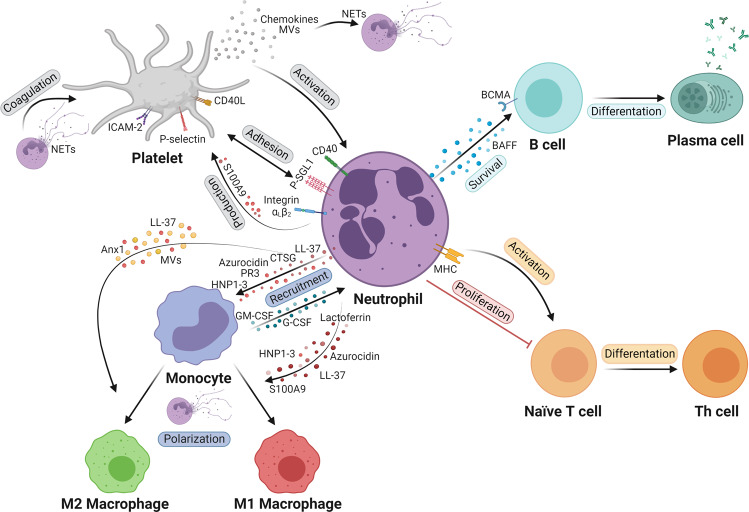
Neutrophil crosstalk with cells in the circulation. Neutrophil interactions with platelets occur by direct adhesion through PSGL-1, integrin α_L_β_2_, and CD40 on neutrophils and P-selectin, ICAM-2 and CD40L on platelets. S100A8/A9 secreted by neutrophils promotes megakaryocyte proliferation and platelet production. Platelet secretion by PEVs containing chemokines and the release of chemokines from platelet granules activate neutrophils and promote NET formation. Conversely, NET proteins activate the coagulation cascade. Neutrophils promote B cell survival and differentiation to plasma cells through the secretion of BAFF, which binds to BCMA on B cells. Neutrophils can act as APCs to promote T cell differentiation in effector T cells through MHC molecules or inhibit T cell proliferation. Monocytes can extend the neutrophil lifespan and promote neutrophil recruitment through the secretion of GM-CSF and G-CSF. Monocyte recruitment is mediated by granule proteins released from neutrophils. Lactoferrin, azurocidin, S100A9, HPN1–3, LL-37 and NETs induce M1 macrophage polarization, while NETs, LL-37 and Anx1 induce M2 polarization. Anx1 annexin 1, APCs antigen presenting cells, BAFF B cell activating factor; BCMA, B cell maturation antigen, CD40L CD40 ligand, HNP1–3 human neutrophil peptides 1–3, ICAM intercellular adhesion molecule, MHC major histocompatibility complex, NET neutrophil extracellular trap, PEVs platelet extracellular vesicles, PSGL-1 P-selectin granulocyte ligand 1

Chronic inflammation is characterized by a prothrombotic state caused by the mutual activation of neutrophils and platelets (Fig. 1). Neutrophil-platelet complexes are found in humans with chronic inflammatory diseases such as inflammatory bowel disease (IBD) [7], psoriasis [8], ulcerative colitis (UC) [9], and atherosclerosis [10]. The interaction with platelets triggers several key neutrophil processes, including adhesion to the endothelium [11] and the formation of NETs [12]. Moreover, platelet numbers are increased in type 2 diabetic mellitus (T2DM) patients by neutrophil S100A8/A9 secretion. S100A8/A9 binding to the receptor for advanced glycation end products (RAGE) expressed on liver-resident Kupffer cells increases thrombopoietin production, which in turn triggers megakaryocyte proliferation and platelet production [13].

After endothelial activation by stimuli such as injuries or pathogen recognition, platelet-neutrophil complexes are formed by the binding of platelet P-selectin with P-selectin granulocyte ligand (PSGL)-1 on neutrophils. This interaction promotes further interactions between intercellular adhesion molecule (ICAM)-2 and CD40 ligand (CD40L) on platelets and integrin α_L_β_2_ and CD40 on neutrophils, which enables the firm formation of platelet-neutrophil complexes [14–16]. In mouse models of psoriasis, blocking neutrophil-platelet interactions by infusion of an anti-PSGL-1 antibody reduces ear thickness in imiquimod-induced psoriasis [8]. In atherosclerotic patients, neutrophil-platelet complexes stimulate the secretion of S100A8 from neutrophils, which binds to Toll-like receptor (TLR)4 and promotes an inflammatory response, resulting in elevated levels of tumor necrosis factor (TNF)α and interleukin (IL)1β and the migration and adhesion of neutrophils [10].

In addition to promoting neutrophil adhesion to the endothelium, crosstalk between platelets and neutrophils leads to NET formation. C-X-C motif chemokine ligand (CXCL)4 released by platelets from patients with anti-neutrophil cytoplasmic antibody (ANCA)-associated vasculitis promotes NET formation [17]. Platelets can bind bacteria on the surface, forming a platelet-bacteria bundle. This structure has been shown to serve as a platform for neutrophil adhesion and NET release in a mouse model of sepsis [18]. Cathelicidin (LL-37/CRAMP), which is released from neutrophils, promotes platelet-leukocyte aggregation and neutrophil recruitment through the induction of platelet activation in thrombi in mice [19]. Inhibiting platelet aggregation with antiplatelet therapy reduces the release of neutrophil-activating substances from α-granules in atherothrombosis [15]. Platelet extracellular vesicles (PEVs) contain CXCL5, which can be transferred to the endothelium and thereby promote neutrophil adhesion, and the PEV surface is composed of different adhesive glycoproteins, such as β1-integrin and P-selectin [20]. PEVs isolated from patients with systemic sclerosis release higher levels of high mobility group box (HMGB) and promote neutrophil activation and NET formation in vitro [21]. A role for NETs in a procoagulant phenotype has been established in IBD, and IBD-derived human NETs increase the risk for thrombosis by enhancing platelet microparticle release through TLR2 and TLR4 in vitro [7].

Platelet-endothelial cell (EC) interactions are crucial for myeloid cell recruitment. Moreover, the interaction between platelets and neutrophils promotes the activation and transmigration of neutrophils to sites of injury, as well as NET formation. Conversely, platelet-neutrophil complexes enhance platelet activation and thrombus formation.

### Neutrophil interactions with adaptative immune cells

Neutrophils interact with B cells by contact-independent signals such as the secretion of B cell activating factor (BAFF). BAFF is involved in the survival, maturation, and differentiation of B cells, is expressed on the neutrophil surface and within intracellular stores and is upregulated in chronic diseases [22] (Fig. 1). Cigarette smoke (CS) is the primary cause of chronic obstructive lung disease (COPD) [23], and neutrophil infiltration in the lungs of wild-type (WT) mice exposed to CS correlates positively with BAFF expression. In vitro, BAFF secretion was increased in neutrophils treated with cigarette smoke extract. Accordingly, the depletion of neutrophils reduces BAFF expression in the lung, and mice that are deficient in BAFF have decreased neutrophil infiltration in the lung [24]. Rheumatoid arthritis (RA) is characterized by the presence of rheumatoid factors and anti-citrullinated protein antibodies (ACPAs) in human serum [25]. Human ACPA-positive B cells stimulated with citrullinated proteins induce neutrophil migration in vitro by secreting CXCL8, a neutrophil chemotactic factor [26], indicating a role for B cell-neutrophil interactions in RA. In addition, LL-37-DNA complexes derived from systemic lupus erythematosus (SLE) patients specifically induce the production of anti-LL-37 antibodies. Hence, autoantibodies against LL-37 and immune complexes of DNA or RNA and autoantibodies can be detected in individuals with this chronic disease [27].

In addition to neutrophil interactions with B cells, myeloid-derived suppressor cells (MDSCs), which are a neutrophil population (see neutrophil heterogeneity in chronic inflammation), can regulate T cells in chronic inflammatory conditions. Thus, skin from psoriatic patients accumulates more MDSCs than skin from healthy donors. Under this chronic condition, MDSCs expand and increase CD4+ T cell differentiation to T helper (Th)17 T cells. Therefore, imiquimod-induced psoriatic mice treated with gemcitabine, an inhibitor of MDSCs that is a nucleoside analog and a chemotherapeutic agent, exhibit reduced MDSC accumulation and infiltration of Th17 and T regulatory cells in the spleen, thus reducing inflammation and disease severity [28]. Furthermore, neutrophils can acquire antigen-presenting cell (APC) properties [29, 30]. In RA, NET peptides are internalized by synovial fluid fibroblasts, loaded in major histocompatibility complex (MHC) II molecules, and presented to T cells. This process promotes T and B cell responses, augmenting the inflammatory response [31].

In summary, the secretion of BAFF from neutrophils promotes B cell survival and proliferation, plasma cell differentiation and antibody production. In chronic diseases such as COPD, SLE or RA, these effects are enhanced, exacerbating the disease through an autoreactive B cell response and autoantibody overproduction. Moreover, neutrophils and MDSCs can inhibit T cell proliferation or promote CD4+ T cell differentiation depending on the disease context (Fig. 1).

### Neutrophil-monocyte/macrophage interplay

Tissue-resident macrophages can be activated and recruit neutrophils to the inflammatory site by secreting chemoattractants such as CXCL1, CXCL2, IL1α and C-C motif chemokine ligand (CCL)2 and increase the neutrophil lifespan by secreting granulocyte-macrophage colony-stimulating factor (GM-CSF), granulocyte colony-stimulating factor (G-CSF) and TNFα [32]. Once at the site of inflammation, neutrophils increase the inflammatory response by recruiting monocytes via the release of LL-37, azurocidin, cathepsin G (CTSG), human neutrophil peptides 1–3 (HNP1–3) and proteinase 3 (PR3) [33, 34]. Neutrophils have been shown to shape monocyte differentiation and macrophage polarization. During infection, azurocidin and lactoferrin released by neutrophils induce the polarization of macrophages to a proinflammatory M1 phenotype. Lactoferrin is taken up by macrophages from apoptotic neutrophils and enhances the microbicidal activity of macrophages [35]. In addition, the neutrophil granule proteins azurocidin and HNP1–3 induce M1 macrophages, which are characterized by TNFα and interferon (INF)γ release and enhanced phagocytosis [36]. The alarmin S100A9 induces the secretion of proinflammatory cytokines and matrix metalloproteinases (MMPs) by synovial macrophages in osteoarthritis [37]. In atherosclerosis, cholesterol crystals induce NET formation, which in turn primes macrophages to produce and release cytokines that activate Th17 cells, resulting in the amplification of inflammation [38]. Moreover, coculturing M2 macrophages and NETs increases the secretion of proinflammatory cytokines by M2 macrophages [39]. In addition, LL-37 can induce an anti-inflammatory and resolution response in acute infections, reducing proinflammatory cytokine secretion without compromising phagocytosis or bacterial clearance [40–42]. In contrast, in chronic inflammation, LL-37 activates macrophages and increases proinflammatory cytokine release, thus inducing M1 macrophage polarization [43]. M2 macrophage polarization can also be facilitated by neutrophil microvesicles (MVs) and is partially mediated by annexin A1 and phosphatidylserine expression on MVs in vitro [44]. The switch from a proinflammatory phenotype to a resolution phenotype enables tissue repair, and disruptions in this switch could lead to chronic inflammation. In the context of infection, dying neutrophils are cleared by macrophages through a process called efferocytosis. This process is dependent on the release of annexin A1 from neutrophils [45], activation of NADPH oxidase by the TLR2/4-MyD88 signaling pathway [46], and neutrophil gelatinase-associated lipocalin (NGAL) [45]. In vitro experiments using human macrophages differentiated with GM-CSF showed M2 macrophage differentiation after treatment with apoptotic neutrophils [47]. However, apoptotic neutrophils induce this switch, and in vitro experiments with human blood neutrophils and monocyte-derived macrophages revealed that viable neutrophils can reduce proinflammatory cytokine release and the nuclear factor kappa B (NF-κB) signaling pathway in lipopolysaccharide (LPS)-induced macrophages [48]. Moreover, in a mouse model of myocardial infarction, annexin 1 enhanced vascular endothelial growth factor-A release from macrophages and thus promoted their polarization to a reparative phenotype [49].

In summary, neutrophils contribute to monocyte recruitment, and tissue-resident macrophages favor neutrophil recruitment. Granule proteins and NETs can enhance the resolution phenotype in macrophages, which contributes to the resolution of inflammation through neutrophil clearance via efferocytosis, pathogen phagocytosis and NET degradation within macrophages. However, neutrophil granule proteins and NETs can promote macrophage activation and proinflammatory cytokine secretion, exacerbating inflammation (Fig. 1). However, monocyte/macrophage-neutrophil interactions in the context of chronic inflammation need to be further investigated.

## Neutrophil heterogeneity in chronic inflammation

Recently, it has become clear that the neutrophil population is not homogenous and is now considered a heterogeneous population. Neutrophil heterogeneity includes phenotypic plasticity, which affects several immunoregulatory neutrophil functions. To study neutrophil heterogeneity, neutrophils can be isolated from peripheral blood by density gradient centrifugation. In healthy donors, normal-density neutrophils (NDNs) are found on top of erythrocytes after density gradient centrifugation. However, patients with acute or chronic inflammation exhibit a heterogeneous population of mature and immature neutrophils in the mononuclear cell fraction. This population is known as low-density neutrophils (LDNs) [50]. The LDN population was first described in SLE and RA [51] and has now been well characterized in both diseases [52, 53]. The LDN population includes granulocytic/polymorphonuclear-myeloid-derived suppressor cells (PMN-MDSCs) with immunosuppressive properties and low-density granulocytes (LDGs), which are characterized as having proinflammatory effects [54, 55]. The PMN-MDSC population has been extensively investigated in cancer patients in whom PMN-MDSCs are expanded compared to healthy subjects. The PMN-MDSC population suppresses T cell proliferation via the release of ROS and arginase 1. Another neutrophil subset found in cancer is tumor-associated neutrophils (TANs), which are neutrophils that have infiltrated tumor tissue and that can exert pro- or antitumor effects [56]. In this review, we will focus on the subsets that have been studied in selected chronic inflammatory disease conditions. For more information about neutrophil heterogeneity in cancer, we refer to other papers [57, 58].

Obese *db/db* mice, which are a model of T2DM, can be infected with *S. aureus* and have higher numbers of LDNs in the blood than control mice and noninfected obese *db/db* mice [59]. Although LDNs were first identified under pathological conditions, LDNs have recently been identified in the blood of healthy donors, representing up to 5% of the total mononuclear cell fraction [60] if artifacts are excluded. Classically, PMN-MDSCs have been described as immunoregulatory cells that suppress T cell proliferation in several diseases, such as cancer, by permitting tumor progression [55]. Suppression by PMN-MDSCs is accomplished by the induction of antigen-specific T cell tolerance, and ROS production is crucial [61]. Although MDSCs have been studied extensively in the field of cancer, recent data have demonstrated that PMN-MDSCs are expanded in patients with T2DM compared to healthy controls [62]. In vitro PMN-MDSCs derived from T2DM patients produce high levels of ROS compared to PMN-MDSCs derived from healthy donors in which ROS production is low. ROS production in patient-derived PMN-MDSCs is aggravated under hyperglycemic conditions [62]. MDSCs in BALF correlated with airflow limitation severity in COPD patients [63], accumulated in the human liver with NAFLD [64], and correlated positively with the clinical parameters alanine transaminase (ALT), aspartate aminotransferase (AST) and globulin [65]. Moreover, *S. aureus* infection in a diabetic mouse model causes an increase in LDN and NET production compared to infected nondiabetic mice [59]. However, more studies are needed to investigate the role of the specific subtypes PMN-MDSCs and LDGs in other chronic diseases, such as NAFLD or IBD.

As neutrophil subset classification is made according to buoyant density, it is important to consider that blood handling and isolation techniques can change NDNs to LDNs. This effect has been shown in healthy donors and can contribute to inaccurate conclusions among studies [54, 66]. Therefore, the development of markers and functional assays are necessary to distinguish between neutrophil populations, but to the best of our knowledge, no consensus has been reached [67]. One attempt to classify neutrophil subsets based on surface marker expression was recently published by Bongers et al. Neutrophils were classified based on the surface expression of CD16 and CD62L. CD16^high^CD62L^high^ neutrophils are found in the circulation during homeostasis. During acute inflammation induced by LPS administration, trauma or infection in humans, CD16^low^CD62L^low^ immature neutrophils are recruited to the circulation from the bone marrow. These cells have higher antibacterial activity than CD16^high^CD62L^high^ neutrophils, are hypersegmented and suppress T cell proliferation. CD16^low^ and CD62L^low^ neutrophils increase during acute inflammatory conditions in patients with trauma or infection or after LPS challenge, while chronic inflammation (COPD and HIV) does not affect circulating levels of CD16^low^ and CD62L^low^ neutrophils [68]. The difficulty in defining neutrophil heterogeneity by gradient density and/or surface markers has led to the use of single-cell sequencing to characterize and define different neutrophil subpopulations in steady-state conditions and during disease. Single-cell RNA sequencing of neutrophils isolated from bone marrow (BM), peripheral blood and the spleen revealed eight neutrophil populations that correspond to BM granulocyte-monocyte progenitor (GMP), proNeutrophil (Neu), preNeu, immature(imm)Neu and mature(m)Neu cells. In peripheral blood, three subsets of mature neutrophils were distinguished (PMNa, PMNb, PMNc) [69]. Recently, the term *“*neutrotime*”* has been established as a spectrum based on neutrophil maturation from the BM to the peripheral blood. Large transcriptional changes were defined during the transition from preNeu to immNeu in the BM and from mNeu in the BM to mature neutrophils from the blood [70]. During inflammation, several mouse models of inflammation, such as arthritis, pneumonitis, peritonitis [70] and after bacterial infection [69], the accumulation of immNeu cells occurred within inflamed tissues. Hence, analysis based on the transcriptome appears to be a more appropriate technique to cover the diversity of neutrophils that other methods.

Although neutrophil populations have been studied in homeostasis as well as several diseases, the data are limited with regard to the contribution of specific subsets in the chronic inflammatory diseases highlighted in this review. Future investigations should include the presence and function of different neutrophil populations in chronic diseases, and studies should take neutrophil heterogenicity into account when designing therapeutic strategies to target or enhance neutrophil functions.

## Neutrophils in chronic inflammation

### Obesity-related diseases

T2DM is a risk factor for developing NAFLD, as it accelerates the progression of liver disease. Approximately 55% of patients with T2DM have NAFLD [71]. Moreover, T2DM and NAFLD have been linked to obesity, which is characterized by the abnormal accumulation of adipose tissue, leading to chronic low-grade inflammation and an imbalance in the immune profile, including the activation of neutrophils [72].

#### Type 2 diabetes mellitus

In 2015, it was estimated that 1 in 11 adults globally (approximately 415 million people) aged 20–79 years, had diabetes, and over 90% of diabetes mellitus cases were T2DM. T2DM is a chronic disease characterized by high levels of glucose in the blood, impaired insulin secretion and/or insulin resistance due to dysregulated carbohydrate, lipid and protein metabolism. Insulin resistance occurs many years before the onset of T2DM and is caused by obesity, physical inactivity and genetic predisposition [73]. Neutrophil activation, which is assessed as the expression of neutrophil elastase (NE) and myeloperoxidase (MPO) in peripheral blood leukocytes [74], NE serum levels [75], or MPO plasma levels [76], is enhanced in obese and T2DM patients compared to lean subjects. Bariatric surgery partially decreases neutrophil activation in patients [75]. Furthermore, obese patients exhibit reduced levels of alpha-1-antitrypsin (A1AT), and the levels correlate negatively with body mass index [77], indicating that neutrophils play a role in the disease progression of T2DM.

**Fig. 2:**
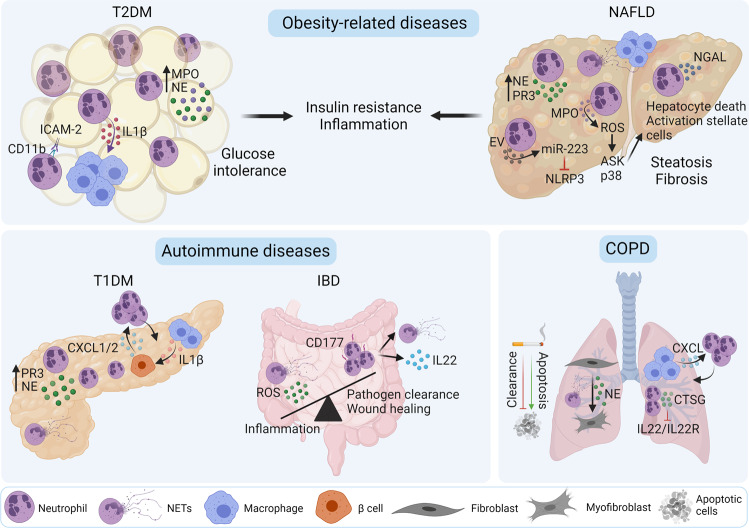
Neutrophil contributions to chronic disease. In obesity-related diseases, adipose tissue is infiltrated by neutrophils, where MPO and NE are released. Neutrophils promote macrophage infiltration through the secretion of IL1β by neutrophil-adipocyte direct interactions via CD11b-ICAM. In NAFLD, neutrophils infiltrate the liver, and ROS production leads to the activation of ASK and p38. The secretion of MPO promotes hepatocyte death and fibrosis, and hepatic NE and PR3 levels increase. miR-223, which is derived from neutrophils, inhibits the NLRP3 inflammasome. NETs lead to increased inflammation by recruiting monocytes/macrophages. These diseases are characterized by insulin resistance and inflammation. The autoimmune disease T1DM is characterized by neutrophil infiltration in the pancreas. β-cells of the islets release CXCL1 and 2, promoting neutrophil recruitment and infiltration. CXCL1 and 2 expression is induced by IL1β, which is secreted by macrophages. Neutrophils release NE and PR3 within the pancreas, increasing their levels. In the context of IBD, neutrophils can act either as beneficial players promoting pathogen clearance and wound healing through IL22 and NETs or as detrimental players enhancing inflammation via PAD4, proteases and ROS secretion. Cigarette smoke, the primary cause of COPD, inhibits apoptotic cell clearance while enhancing cell apoptosis. Chemokines released by alveolar macrophages induce neutrophil recruitment to the lung. NE and NETs trigger the transition of fibroblasts to myofibroblasts, leading to increased fibrosis. In addition, CTSG inhibits the IL22/IL22R pathway, increasing infection propensity. ASK apoptosis signal-regulating kinase, CTSG cathepsin G, CXCL CXC chemokine ligand, ICAM intercellular adhesion molecule, IL: interleukin, MPO myeloperoxidase, NAFLD nonalcoholic fatty liver disease, NE neutrophil elastase, NLRP3 NOD-, LRR- and pyrin domain-containing protein 3, PAD4 peptidyl arginine deiminase 4, ROS reactive oxygen species, T1DM type 1 diabetes mellitus

Neutrophil infiltration and the consequent release of granule proteins in adipose tissue in mice fed a high-fat diet (HFD) contribute to the development of insulin resistance. Insulin signaling is impaired by nitration of the β subunit of the insulin receptor by MPO [78] and the degradation of insulin receptor substrate 1 by NE [79]. Therefore, mice with NE deficiency [77, 79] or MPO deficiency [78] have reduced adipose tissue inflammation and improved carbohydrate metabolism, including improved glucose tolerance and insulin sensitivity and a reduction in insulin resistance. Neutrophil effects can also be mediated by direct interaction with adipocytes. Neutrophil adhesion to adipocytes is mediated by the interaction of CD11b with ICAM-1 on adipocytes in vitro [80]. Adhesion results in increased IL1β expression via the NF-κB pathway in neutrophils and contributes to the expression of chemotactic molecules and the infiltration of macrophages, amplifying adipose tissue inflammation and subsequently the occurrence of insulin resistance [81]. In summary, neutrophils infiltrate adipose tissue in obesity and T2DM. Within adipose tissue, neutrophils secrete NE and MPO, and in mouse models of T2DM, these factors are thought to promote the development of insulin resistance and inflammation in adipose tissue (Fig. 2). Future investigations in patients need to be performed to examine the contribution of neutrophil protease to T2DM progression, especially with regard to MPO as a possible target. MPO deficiency in humans has been shown to increase susceptibility to fungal infections in some patient groups but does not affect inflammatory disease progression [82].

#### Nonalcoholic fatty liver disease

NAFLD is the predominant chronic liver disease worldwide and is the most common cause of mortality and morbidity associated with liver disease [83]. NAFLD involves different liver conditions, including nonalcoholic fatty liver characterized by steatosis and nonalcoholic steatohepatitis (NASH) with inflammation, hepatocyte damage and fibrosis as the main features [83]. Several studies in humans and mice have pointed out a role for neutrophils in NAFLD. Neutrophil infiltration in the liver promotes NASH development [84], and circulating neutrophils correlate with severity in NASH patients [85].

Modulating key targets in neutrophil recruitment to the liver results in reduced disease progression. G-CSF-deficient mice fed a HFD exhibit reductions in neutrophil and macrophage infiltration in the liver, alleviating NAFLD progression [86]. Myeloid-p38γ/δ-deficient mice are protected against fatty liver development and glucose intolerance through reduced neutrophil infiltration in the liver. Consistent with these results, patients with obesity and NAFLD have increased liver p38δ expression [87]. Moreover, tail vein injection of an adenoviral vector that overexpresses CXCL1, a chemokine implicated in neutrophil recruitment, results in enhanced neutrophil accumulation in the liver in mice fed a HFD. Reactive oxygen species (ROS) produced by recruited neutrophils activate the liver stress kinases apoptosis signal-regulating kinase 1 (ASK1) and p38 and promote the progression to NASH. Intraperitoneal injection of IL22-Fc, a cytokine that protects the epithelium, in mice overexpressing CXCL1 alleviates this condition by reducing inflammation, liver injury and fibrosis [88]. NGAL induces C-X-C motif chemokine receptor (CXCR)2 expression on neutrophils and results in their subsequent accumulation in the liver. Hence, *Ngal* deficiency in *apolipoprotein(Apo)e*^*−/−*^ mice decreases neutrophil and macrophage infiltration in the liver compared to that in *Apoe*^*−/−*^ mice. Accordingly, human plasma levels and hepatic expression of NGAL are increased with disease severity [89]. Neutrophil infiltration and NE levels in the livers of WT mice peak during the day and correlate with *aryl hydrocarbon receptor nuclear translocator like* (*Arntl)/Bmal1*, a clock gene that is thought to induce lipogenesis. In fact, lipid accumulation was increased during the day in WT mice fed a HFD. Neutrophils promote the activation of c-Jun N-terminal kinases (JNKs), reducing the hepatic expression of *fibroblast growth factor 21*. Therefore, neutrophil depletion in mice diminishes the expression of *Arntl/Bmal1* and circadian locomotor output cycles kaput (clock) and reduces the overall activation of JNK, reducing hepatic steatosis [90].

Plasma concentrations of the neutrophil granule proteins PR3 and NE correlate positively with disease severity in NAFLD and T2DM patients, and A1AT levels are reduced, producing an imbalance in NE/A1AT, which is a ratio associated with NASH progression [91, 92]. Furthermore, the neutrophil-derived proteins NE, PR3 and CTSG, as well as caspase 1 and 11, contribute to liver steatosis in mice fed a HFD [93, 94]. A reduction in steatosis and inflammation was also demonstrated in NE-deficient mice fed a Western diet, in part through reducing the hepatic expression of ceramides and serine palmitoyltransferase subunit 2, an enzyme associated with de novo synthesis of ceramides [95]. Treatment with the protease inhibitor A1AT decreased liver lipid accumulation in WT mice fed a HFD compared to phosphate-buffered saline (PBS)-treated WT mice fed a HFD [93]. Pharmacological inhibition of NE by sivelestat diminished liver inflammation in *Apoe*^*−/−*^ mice fed a HFD [96]. In addition to neutrophil delivery of proteases in the liver, neutrophil-derived microRNAs also contribute to disease progression. In HFD-fed mice, miR-223, which is packaged in extracellular vesicles, is transferred to hepatocytes via low-density lipoprotein receptor and APOE, leading to the inhibition of NOD-, LRR- and pyrin domain-containing protein 3 (NLRP3) gene expression, which is involved in NAFLD progression [97].

In addition to NE, plasma MPO levels are also higher in NASH patients [98] and mouse models of NASH [99]. In mice, MPO induces hepatocyte death and the activation of hepatic stellate cells, which are collagen-producing cells that contribute to fibrosis. In vitro, oxidative products derived from MPO activate tumor growth factor (TGF)β, and in vivo treatment of mice with a TGFβ inhibitor reduced steatosis and fibrosis. Hence, MPO deficiency in mice diminishes hepatocyte injury by reducing hepatic stellate cell activation, resulting in decreased TGFβ levels and fibrosis [100]. Additionally, *low-density lipoprotein receptor* (*Ldlr)*^*−/−*^*Mpo*^*−/−*^ mice fed a HFD had reduced hepatic neutrophil, lymphocyte, lipid, and proinflammatory cytokine levels and fibrosis. These mice also display reduced visceral adipose tissue inflammation [99].

MPO-DNA levels, which are a marker of NETs, are elevated in NASH patients and mouse models of NASH [101, 102]. NETs derived from NASH patients increase thrombus formation and can induce a procoagulant phenotype in ECs [101]. In a mouse model of NASH, steatosis developed independently of NETs, which indicates that NET formation occurs as a consequence of fatty liver accumulation. In this early stage, when only steatosis is present, neutrophil infiltration and NET formation lead to later macrophage infiltration and the production of proinflammatory cytokines. Hence, when NETosis is inhibited, a reduction in inflammation is observed, including a decrease in macrophage and neutrophil levels in the liver. The progression to hepatocarcinoma is also reduced after NETosis inhibition in mice [102]. Similarly, the depletion of neutrophils by antibody injection reduced liver inflammation in WT mice fed a HFD [103] and *Apoe*^*−/−*^ mice fed a methionine-choline-deficient diet [96]. The coculture of CD4+ and CD8+ T cells with NASH-derived neutrophils suppressed the proliferation of these T cells and promoted the activation of neutrophils [85].

Neutrophils and NETs accumulate in the liver in NAFLD patients. In mice, NE, PR3 and CTSG were associated with increased steatosis, inflammation, and insulin resistance, while MPO promoted the development of fibrosis through the activation of hepatic stellate cells (Fig. 2). In combination, these mechanisms could lead to enhanced development and progression of liver disease. Therefore, by reducing neutrophil recruitment and infiltration or inhibiting neutrophil proteases in mouse models, NAFLD progression can be reduced. To determine the potential role for neutrophil proteases in the development of NAFLD in humans, future studies need to be performed.

### Atherosclerosis

Cardiovascular disease (CVD) is the leading cause of mortality worldwide [104]. The underlying pathological process of CVD is atherosclerosis, a slowly progressing chronic disorder of large and medium-sized arteries that is characterized by the accumulation of lipids in the arterial wall, the infiltration of immune cells, and the formation of a fibrous cap composed of smooth muscle cells and collagen [105]. Neutrophils are implicated in the development and progression of atherosclerosis, which has been reviewed elsewhere [106–108]. However, as the field is continuously expanding, this review will summarize discoveries reported in 2020 and 2021.

The *Apoe*^*−/−*^ mouse is a hyperlipidemic mouse strain on a C57BL/6 J background that is commonly used to study the pathophysiology of atherosclerosis [109]. Wild-type mice are protected against atherosclerosis development, as most cholesterol is carried in high-density lipoprotein (HDL) particles, which ensures cholesterol elimination through the reverse cholesterol transport pathway. Therefore, this transgenic mouse model was developed to study atherosclerosis pathogenesis. Genetic deletion of *Apoe* increases plasma cholesterol levels to the range of 400–600 mg/dL, triggering spontaneous atherosclerosis development at approximately 20 weeks of age. High-fat and high-cholesterol diets accelerate atheroma plaque formation as plasma cholesterol levels reach more than 1000 mg/dL [109, 110]. Although this mouse is the most frequently used strain to study atherosclerosis, its pathophysiology differs from that of humans. For example, human lesions occur mainly in coronary arteries, carotids and peripheral vessels, while in mice, atheroma plaques occur in the aortic root, aortic arch and innominate artery [111]. Therefore, the results from mouse studies cannot be directly translated to humans.

One mechanism by which neutrophils contribute to atherosclerosis is through NET formation. LPS administration to *Apoe*^*−/−*^ mice produced larger lesion sizes with the accumulation of myeloid cells and NETs. NET-associated histone 2a is responsible for monocyte adhesion to NETs in a charge-dependent manner [112]. Unstable plaques in patients have increased levels of peptidyl arginine deiminase 4 (PAD4) compared to carotid plaques with stable features [113]. Additionally, coronary thrombi in patients with ST-segment elevation myocardial infarction have increased NETs [114]. Downstream plaque regions with vulnerability features have a major numbers of neutrophils and increased expression of NE and citrullinated histone 3 (citH3) compared to upstream regions [115]. Serum autoantibodies against ApoA1, the main protein fraction of high-density lipoproteins, are a predictor of cardiovascular events. In patients with anti-ApoA1-positive serum, the citH3 signal does not colocalize with neutrophils, suggesting increased production of citH3 by neutrophils [115].

Diabetes can induce or accelerate atherosclerosis, and diabetic characteristics such as higher glucose levels, dyslipidemia and chronic inflammation are involved in atherosclerosis development [116]. Hence, patients with both diabetes and atherosclerosis have been shown to have increased circulating markers of NET formation compared to patients with only atherosclerosis [117]. In a model that mimics transient intermittent hyperglycemia in *Apoe*^*−/−*^ mice, hyperglycemia increases lesion size, myelopoiesis and circulating monocytes and neutrophils compared to those in control mice. S100A8/A9 released by neutrophils in transient intermittent hyperglycemic mice induces myelopoiesis through RAGE activation [118]. Impaired resolution of atherosclerosis in diabetic mice correlates with increased NETs, CD68+, NLRP3+ and caspase 1+ cell numbers, necrotic cores, and plaque sizes compared to those of nondiabetic mice. These parameters are reduced by treatment with DNase 1 [119].

In mice, neutrophil-derived MVs are present in very small amounts under homeostatic conditions but increase as a result of a HFD. In *Apoe*^*−/−*^ mice fed a HFD, MVs are localized in atheroprone sites, such as the inner curvature of the aortic arch where they adhere to and are internalized by ECs [120]. Within ECs, MVs release the miR-155, which is found in atheromatous plaques in *Apoe*^*−/−*^ mice and humans [121], resulting in monocyte recruitment and the acceleration of atheroma plaque formation [120]. Moreover, mice deficient in miR-146a, which inhibits the NF-κB signaling pathway, have increased NET formation in atheromatous plaques and have increased rates of NETosis, increased levels of plasma NE and citH3, increased ROS production, and a procoagulant phenotype in response to LPS injection. Consistent with the results obtained in mice, septic patients exhibit higher plasma levels of DNA-citH3 than nonseptic patients [122].

### Autoimmune diseases

Autoimmune diseases are a varied group of disorders in which damaging immune responses to self-antigens occur. Approximately 3–5% of the population is affected by these diseases, and this percentage is continuously increasing [123]. Autoimmune diseases include several diseases, such as type 1 diabetes mellitus (T1DM), RA, multiple sclerosis, SLE and IBD. Neutrophils have been implicated in all of these conditions, but we will focus on T1DM and IBD. Neutrophils and RA [124], multiple sclerosis [125, 126] or SLE [127] have recently been reviewed elsewhere.

#### Type 1 diabetes mellitus

T1DM is a chronic autoimmune disease characterized by the destruction of insulin-producing β-cells in the islets of the pancreas, which leads to insulin deficiency and a hyperglycemic state. This disease is a combination of different immunologic, genetic and physiologic events, but the exact mechanism is still unclear [128, 129].

Nonobese diabetic (NOD) mice are used as a model to study the pathophysiology of T1DM, and this model shares many similarities with human disease. NOD mice spontaneously develop autoimmunity and T1DM with pancreatic infiltration of innate immune cells at 3 weeks of age and later infiltration of CD4+ and CD8+ T cells at 4–6 weeks of age [130]. Similar to what happens in human disease, NOD mice exhibit autoantibodies and autoreactive circulating T cells, as well as β cell loss and/or dysfunction. Moreover, the locus that accounts for most of the risk in mice is MHC class II, which resembles human leukocyte antigen (HLA) in humans. A limitation of this model of human diabetes is that in NOD mice, the initial antigen is insulin, and the appearance of insulitis is more aggressive and severe, while in humans, different antigens are responsible for T1DM initiation, and insulitis is less severe [131].

Neutrophil infiltration [132, 133] and NE concentrations in the pancreas [132] are increased in neonatal NOD mice compared to control mice. The recruitment of neutrophils to the pancreas at this early age is caused by the expression of CXCL1 and CXCL2 by β-cells and macrophages, and this expression is induced by macrophage IL1β secretion [133]. NETosis contributes to disease progression [134], and immune cell infiltration in the pancreatic islets of NOD mice results in higher levels of citrullination than in control islets [135]. Consequently, inhibiting NE [132] or PADs [135] and the use of a CXCR2 antagonist [133] alleviate the development of diabetes in NOD mice.

Inhibiting NE by sivelestat or elafin [132] or NETs by BB-Cl-amidine treatment [135] in NOD mice resulted in the attenuation of immune cell invasion in the pancreas, a less aggressive T cell response and a more favorable environment for immune tolerance. NET formation is also inhibited by oral staphylococcal nuclease (SNase) delivery by *Lactococcus lactis*. NOD mice that received SNase exhibited decreased NET formation, reduced pancreatic inflammation, increased β-cell mass and enhanced glucose tolerance compared to control mice [136]. Neutrophils derived from T1DM patients are more prone to produce NETs after stimulation and have increased expression of PAD4 [137]. In vitro, NETs isolated from these patients induce the production of IFNγ by CD4 + and CD8 + T cells, which is mediated by monocyte-derived dendritic cells and characterized by lower levels of the antimicrobial peptides MPO and LL37 than NETs isolated from control subjects [138].

Subjects with a high risk of developing T1DM and newly diagnosed T1DM patients have lower circulating levels of neutrophils [139–142], which correlate with reduced serum levels of NE and PR3 [143] and faster disease progression [142, 144]. Lower levels circulating neutrophils are attributed to increased infiltration in the pancreas [141, 142]. The correlation between disease progression and neutrophil counts has been shown to be age-dependent [142], which might explain studies that have shown increased levels of NET-associated proteins [145] and circulating levels of NE and PR3 [146] in newly diagnosed T1DM patients. Neutrophil counts are thus decreased in the circulation of T1DM patients but increased in the pancreas. In the pancreas, neutrophils release proteases and NETs, promoting an inflammatory response (Fig. 2).

#### Inflammatory bowel disease

IBD includes Crohn´s disease and UC. The diseases differ with regard to the inflammatory site. Crohn´s disease can affect any zone of the gastrointestinal tract, whereas UC is restricted to the colon. Several factors contribute to the development of these diseases and are a combination of genetic susceptibility, environmental factors, microbiota and immune system response [147]. The hallmark of active IBD is the infiltration of neutrophils in the intestine, but whether these cells play a protective or harmful role is still unclear.

To study IBD in mice, the most widely used model is dextran sulfate sodium (DSS)-induced colitis. The administration of 1–5% DSS in drinking water produces severe colitis that resembles human disease and is characterized by diarrhea, ulcerations, intestinal inflammation, body weight loss and shortening of colon length. DSS damages the epithelial layer of the large intestine, permitting the dissemination of proinflammatory factors to the tissue. Variations in DSS concentrations and administration frequency allow for the modeling of acute and chronic intestinal inflammation. Although disease progression has several similarities with that in humans, there are differences, including the lack of T and B cell contributions to the development of the disease in this mouse model [148, 149].

In DSS-induced colitis mice, there was evident cell-free DNA and NET formation and deposition [7, 150] and elevated PAD4 expression in the colon [150]. In these studies, DNase 1 treatment or neutrophil depletion with anti-Ly6G antibody decreased colitis development and colitis-associated tumorigenesis [7], and treatment with streptonigrin, a PAD4 inhibitor, diminished citH3 levels in the intestine and colon inflammation [150]. Similar to the mouse model, patients with UC have elevated expression of PAD4 and increased levels of NE, MPO and citH3 in the intestinal mucosa. Neutrophils derived from UC patients produce increased amounts of NETs as a response to TNFα treatment. PAD4 in the affected mucosa and enhanced NET production are thought to augment intestinal inflammation [150].

In addition to the contribution of NETs, neutrophil production of ROS, which is modulated by MAPK-activated protein kinase 2, has been shown to contribute to DSS-induced colitis in mice [151]. In general, neutrophils from IBD patients release more NETs that localize in the colon, and NET degradation is impaired compared to neutrophils from healthy subjects [7]. Monocyte chemotactic protein 1-induced protein 1 (MCPIP1) is highly expressed in neutrophils from the inflamed colons of IBD patients and reduces neutrophil migration and the release of MPO, ROS and proinflammatory cytokines from neutrophils [152]. Although neutrophils isolated from IBD patients produce proinflammatory cytokines [152], in general, the amount of proinflammatory cytokines produced by neutrophils in vitro is lower than that produced by other leukocyte populations, such as monocytes/macrophages [153].

CD177, which regulates endothelial transmigration [154], is highly expressed in circulating neutrophils and the mucosa of IBD patients. Specifically, CD177 + cells produce lower levels of proinflammatory cytokines and have higher bactericidal activity than other cells, suggesting a protective role of this neutrophil subset. Accordingly, colitis mice that are deficient in CD177 + neutrophils have a compromised intestinal barrier and increased colitis development [155]. The stimulation of triggering receptor expressed on myeloid cell (TREM)−1 with an agonistic antibody induced CD177 + neutrophils that produced NETs and IL22 and induced pathogen clearance and wound healing, alleviating colitis [156]. In addition, neutrophils could maintain intestinal barrier homeostasis through TGFβ1 [157] and IL22 secretion [158]. TGFβ1 promotes amphiregulin production by intestinal epithelial cells [157], whereas IL22, which is produced in response to IL23 and TNFα, enhances the secretion of antimicrobial peptides such as S100A8/A9 [158], resulting in the restoration of epithelial barrier function and the resolution of colitis. Therefore, neutrophil depletion or the inhibition of neutrophil adhesion by means of anti-L-selectin aggravates dinitrobenzene sulfonic acid-induced colitis [159].

These studies indicate that neutrophil infiltration in the colon and intestine contributes to inflammation in IBD. IBD patients exhibit increased NET release, PAD4 expression and protease levels. However, specific intestinal and colonic neutrophils are crucial for maintaining intestinal barrier function (Fig. 2). The dual role of neutrophils in IBD [160] requires further investigation to determine if neutrophil function is situation dependent.

### Chronic obstructive lung disease

COPD is a major cause of chronic morbidity and was the third leading cause of death worldwide in 2015. The primary cause of COPD is exposure to CS, but other factors, such as air pollution or fumes, have been implicated. The main features of this disease are airflow limitation, emphysematous alveolar wall destruction, local and systemic chronic inflammation of the airways, alveoli and microvasculature, and recurrent infections. Overall, these disease components result in lung failure and ultimately death. Bacterial and viral infections exacerbate inflammation, hypoxia and airway damage in this disease [23, 161, 162].

To study COPD in mice, the most frequently used model is exposure to CS, as CS is the most important risk factor for human COPD. However, the results between studies are difficult to compare because the type of cigarettes and their composition, the exposure method (whole body or nose-only), and the dose differ [163]. CS exposure models have several similarities with human pathology, such as an increase in lung inflammation and airway fibrosis. However, exposed mice do not produce excessive mucus, periods of exacerbation are absent, and only mild emphysema and modest remodeling of the pulmonary microvasculature occur [163–165].

In vivo acute cigarette smoke exposure inhibits the clearance of apoptotic cells, including alveolar macrophages and neutrophils, in a reversible manner, whereas chronic cigarette exposure irreversibly inhibits efferocytosis, which contributes to the development of COPD [166]. In fact, cigarette smoke exposure induces cell death in neutrophils with the characteristics of apoptosis, autophagy and necrosis in vitro [167].

One of the main features of COPD is neutrophilic inflammation, which correlates with airway obstruction [168] and peripheral airway dysfunction [169]. Infiltration of neutrophils in the lungs is driven by chemotactic factors such as CXCL1, CXCL5, leukotriene B4 and CXCL8, which are produced by epithelial cells and alveolar macrophages [170, 171]. In vitro studies using air-liquid interface cell cultures showed that COPD airway epithelia exhibited enhanced chemotactic activity. Recombinant human club cell secretory protein can neutralize CXCL8 and thereby prevent neutrophil recruitment to the airways [172]. The CXCR2 antagonist MK-7123 improved lung function in patients with moderate to severe COPD, but long-term treatment was associated with a reduction in circulating neutrophils and a slightly increased rate of infections [173].

In healthy people, epithelial lung injury can be caused by infections, inflammation, or exposure to cigarette smoke when neutrophils migrate to the airspaces across the epithelium. The epithelium is repaired through epithelial cell proliferation through neutrophil-mediated activation of the β-catenin pathway, which targets cell proliferation genes in epithelial cells in the lung [174]. In COPD, this repair process is impaired, which contributes to chronic inflammation [175]. Myofibroblasts, which are key cells in the wound healing process, undergo programmed cell death to restore normal tissue architecture and function under homeostatic conditions when the healing process is complete. When this process is dysregulated, the deposition of excessive extracellular matrix and destruction of lung architecture contribute to decreased lung function and tissue pathology [176]. NETs and NE promote the differentiation of lung fibroblasts to myofibroblasts [177, 178]. NE deficiency in a mouse model of idiopathic pulmonary fibrosis results in a reduced number of myofibroblasts [177]. Additionally, in patients with fibrotic interstitial lung disease, NETs are detected close to α-smooth muscle actin-expressing fibroblasts. Experiments with human-derived neutrophils have revealed that myofibroblasts treated with NETs gain a fibrotic phenotype characterized by increased collagen production and proliferation/migration [178]. Similarly, the severity of COPD correlates with sputum NET complexes, and the capacity for phagocytosis is reduced in patients with higher levels of NETs in the sputum [179]. NETosis is induced by the infection of neutrophils with *H. influenzae* and is thought to be a mechanism for IL6 receptor shedding from neutrophils in COPD. Furthermore, in bronchoalveolar lavage fluid (BALF) from patients with COPD, the levels of shed IL6 receptor positively correlate with markers of NETs [180].

Recurrent pulmonary infections are a hallmark of COPD. In patients with COPD, blood neutrophil counts are increased during exacerbations and are related to long-term mortality. Patients with high blood neutrophil counts also have decreased microbiota diversity [181]. Moreover, NE levels in the sputum [182] and *NE* mRNA expression in the peripheral blood mononuclear cells of COPD patients [183] are elevated during COPD exacerbations. Similarly, sputum MPO levels also increase during exacerbation in COPD patients [184]. Therefore, treatment with an MPO inhibitor resulted in decreased oxidative stress and halted emphysema progression and small airway remodeling in guinea pigs exposed to cigarette smoke [185]. Patients with COPD who smoke have elevated serum levels of MMP9, NGAL and proMMP9/NGAL compared to nonsmokers with COPD [186]. In another set of COPD patients, the expression levels of MMP12 and tissue inhibitor of metalloproteases (TIMP) 4 were elevated, and MMP12 levels correlated with the severity of airflow limitation [183]. MVs derived from leukocytes present in the BALF of COPD patients were characterized and showed that neutrophil MV levels were higher in patients than in control individuals and that the presence of MVs correlated with COPD severity [187].

Endogenous antimicrobial defense is fundamental in overcoming an infection, and IL22/IL22R signaling is involved in host defense. However, in COPD, proteases derived from neutrophils, such as CTSG, have been shown to cleave IL22R, disrupting the signaling pathway, reducing the antimicrobial effects and facilitating bacterial infections. In addition, the release of these proteases is enhanced after cigarette smoke exposure, and the products generated by proteolysis serve as proinflammatory stimuli [188] that exacerbate the inflammatory condition.

In summary, patients with COPD have elevated levels of neutrophils in circulation and the lung. Within the lung, neutrophils release NETs and NE, which promote the transition from fibroblasts to myofibroblasts, and CTSG, which inhibits the IL22/IL22R pathway. Overall, these factors promote lung fibrosis, tissue destruction and the disruption of antimicrobial defense (Fig. 2).

### Therapeutic implications and future considerations

The studies summarized in the previous section show that the contribution of neutrophils to chronic disease is significant. Although the pathology of the chronic diseases described here varies greatly, the role of neutrophils in these diseases has common features, such as protease release, NET formation and impacts on other immune cells. Therefore, it is tempting to consider targeting neutrophil function as a strategy to alleviate signs and symptoms of disease or to halt the low-grade systemic inflammation that creates a vicious cycle that worsens the chronic condition. Based on neutrophil biology, it is possible to limit their effects by disrupting several steps, such as mobilization from the bone marrow, recruitment to the tissue, release of proteases and formation of NETs. Table 1 summarizes targets and inhibitors used to interfere with neutrophil function in atherosclerosis, T1DM and T2DM, COPD, NAFLD and IBD, the disease models and the results of the studies. Most studies are in the experimental and preclinical phase, and the summary serves as a direction for future investigations with regard to side effects and possible use in patients.

**Table 1:** Therapeutic approaches to target neutrophils in chronic diseases

Neutrophil functionality	Target and inhibitor	Chronic disease	Experimental set up	Main result	Reference
Recruitment	CXCR2 antagonist SB225002	T1DM	NOD mice	Reduced recruitment of neutrophils from blood to pancreas attenuated development of disease.	[133]
	Daily oral administration of CXCR2 antagonist MK-7123	COPD	Patients with moderate to severe COPD	Increased lung function (assessed by FEV1), reduction in sputum neutrophils. Decrease in circulating neutrophils and slight increase in total and respiratory infections with long-term treatment.	[173]
	IL-8 (CXCL8) neutralization with rhCCSP	COPD	In vitro air-liquid interface cell culture	Reduced neutrophil chemotaxis.	[172]
	CCR2 antagonist (RS102895) daily at ZT17	Atherosclerosis	Apoe−/− mice fed a high-fat diet	Reduced arterial myeloid cell adhesion, atherosclerotic lesion formation and macrophage accumulation.	[201]
	Canakinumab (monoclonal antibody targeting IL-1β)	Atherosclerosis	Randomized trial in patients with prior history of MI and high-sensitivity C-reactive protein	Reduction in cardiovascular events.	[204, 205]
	Genetic deletion of G-CSF	NAFLD	Mice fed high-fat diet	Deficiency in G-CSF resulted in attenuated insulin resistance and hepatic steatosis.	[86]
Degranulation	NE was inhibited by sivelestat or elafin	T1DM	NOD mice	Inhibition of NE attenuated macrophage infiltration and T-cell mediated destruction of pancreatic beta-cells resulting in reduction in spontaneous development of disease.	[132]
	Genetic deletion of MPO Inhibition of MPO by 4-aminobenzoic acid hydrazide administered ip	T2DM	Mice fed high-fat diet	Deficiency in or inhibition of MPO improved insulin sensitivity.	[78]
	NE was inhibited by sivelestat administered intraperitoneally	NAFLD	Apoe^−/−^ mice fed a high-fat, high-cholesterol diet	Alleviation of metabolic syndrome components including NASH-associated inflammation.	[96]
	Genetic deletion of NE Daily oral administration of NE inhibitor GW311616A	T2DM	Mice fed high-fat diet	Deficiency in of inhibition of NE improved insulin sensitivity and decreased adipose tissue inflammation.	[77]
	Genetic deletion of NE	T2DM	Mice fed high-fat diet	Deficiency in NE decreased adipose tissue inflammation and reduced insulin resistance.	[79]
	Genetic deletion of NGAL	NAFLD	Mice fed high-fat, high-cholesterol diet	Deficiency in NGAL alleviated hepatic injury, inflammation and infiltration of neutrophils.	[89]
	Genetic deletion of NE	NAFLD	Mice fed Western diet	Deficiency in NE resulted in reduced inflammation of the liver.	[95]
	Genetic deletion of MPO	NAFLD	Ldlr^−/−^ mice fed high-fat diet	Deficiency of MPO resulted in reduced hepatic fibrosis and adipose tissue inflammation.	[99]
NET formation	PAD inhibition by BB-Cl-amidine	T1DM	NOD mice	Reduced levels of citrullination in pancreas prevented development of disease.	[135]
	DNA degradation by SNase delivered orally through Lactococcus lactis	T1DM	NOD mice	Decrease in serum NETs, NE and PR3. Treatment resulted in delayed onset of disease.	[206]
	PAD inhibition by Cl-amidine oral administration daily	IBD	TNBS-induced colitis in mice	Reduction of NETs in colon. Alleviation of clinical colitis index and tissue inflammation.	[151]
	DNA degradation by oral administration of Staphylococcal nuclease (SNase/ ALG-SNase)	IBD	DSS induced UC in mice	Decreased NETs in colon, alleviation of clinical signs and tissue inflammation.	[207]
	DNA degradation by iv injection of DNase I	IBD	DSS induced UC in mice	Treated mice were protected from colitis as well as thrombus formation and platelet activation.	[7]
	Anti-TNF-alpha (infliximab) infusion PAD4 was inhibited by ip administration of streptonigrin	IBD	Patients with UC DSS induced UC in mice	Reduction in PAD4-positive cells and NET-associated proteins. Reduction in colonic inflammation.	[150]
	PAD4 inhibitor in collagen IV directed nanoparticles	Atherosclerosis	Apoe^−/−^ mice	Inhibition of PAD4 resulted in attenuated endothelial injury in a mouse model of superficial erosion.	[202]
	Daily subcutaneous administration of Cl-amidine or genetic deletion of PAD4 to inhibit NET release. Intraperitoneal injection of anti-histone H4	Atherosclerosis	Apoe^−/−^ mice fed a high fat diet, model for unstable plaque	Blockade of NET release (pharmacological or genetic) and specific inhibition of histone H4 reduced plaque vulnerability.	[208]
	Genetic deficiency in PAD4 or DNase I treatment	Atherosclerosis	Ldlr^−/−^ mice fed modified high fat diet (1.25% cholesterol), model for unstable plaque	Decreased intimal permeability and protection of intimal integrity.	[209]
	Inhibition of PAD4 by daily subcutaneous injections with Cl-amide	Atherosclerosis	Apoe^−/−^ mice fed a high fat diet	Reduction in atherosclerotic lesion area and protection from arterial thrombosis.	[210]
	Therapeutic anti-citrullinated protein antibody (tACPA) to inhibit NET formation	IBD	DSS-induced colitis in mice	Decreased colon inflammation	[211]
	Genetic deficiency in PAD4 or DNase I treatment	NAFLD	NASH induced by neonatal streptozotocin and high-fat diet in mice	Inhibition of NET formation did not reduce fatty liver development, but resulted in reduced hepatocarcinoma.	[102]

Inhibitors are sorted by neutrophil functionality: recruitment, degranulation and NET formation. Experimental models and main findings are provided for each reference

*T1DM* summary of neutrophil targets in the chronic inflammatory disease type 1 diabetes, *IBD* inflammatory bowel disease, *T2DM* type 2 diabetes, *NAFLD* nonalcoholic fatty liver disease, *COPD* chronic obstructive pulmonary disease and atherosclerosis

In addition to directly targeting neutrophil recruitment or neutrophil effects in the tissue, it is possible to interfere with other immune cells that promote neutrophil recruitment through cytokine production. The excessive neutrophil infiltration seen in some chronic inflammatory conditions can generate a positive feedback loop, as recruited neutrophils produce chemoattractants, which leads to even more recruitment. Interference with neutrophil accumulation using a CXCR2 blocking antibody improved clinical scores in mouse models of RA and atopic dermatitis [189]. However, in a study of patients with COPD, the reversible CXCR2 antagonist danirixin did not yield beneficial results [190].

Considering neutrophil function in acute inflammation, one strategy to interfere with neutrophils would be to promote proresolving effects. By promoting the resolution of inflammation by reducing neutrophil infiltration, producing proinflammatory mediators and enhancing the clearance of cellular debris and apoptotic cells, it could be possible to halt some chronic inflammatory conditions. The importance of inflammation resolution in aging was highlighted in a study using mice lacking lipoxin A4/formyl peptide receptor 2 (ALX/FPR2). Following myocardial infarction, FPR2-deficient mice had decreased numbers of resolving macrophages and increased numbers of neutrophils at the site of injury, indicating that FPR2 could play an important role in resolution following ischemic injury [191]. Resolvin E1 (RvE1), an anti-inflammatory lipid mediator derived from the ω-3 fatty acid eicosapentaenoic acid, promotes phagocytosis-induced apoptosis in neutrophils in vitro and in mouse models of acute lung injury [192]. Obese individuals with higher expression of the receptor for RvE1 in adipose tissue have attenuated adipose tissue inflammation, increased liver insulin sensitivity and reduced levels of proinflammatory cytokines in the circulation. Genetic deletion of the receptor in mice is associated with increased adipose tissue and liver inflammation [193]. Conversely, weight loss in patients with metabolic syndrome leads to an enhanced ability of neutrophils to release RvE1 [194]. Dietary supplementation with eicosapentaenoic acid in mice fed a HFD resulted in improved glycemic control partially through RvE1 [195] and decreased atherosclerotic plaque formation in *Apoe*^*−/−*^ mice fed a Western diet [196]. The data with regard to supplementation in patients vary. For example, patients with elevated triglycerides who received icosapent, a highly purified eicosapentaenoic acid ethyl ester, had a reduced risk of cardiovascular events [197]. A meta-analysis of large trials investigating the correlation between omega-3 fatty acid supplements and cardiovascular disease showed that supplementation had no positive effect on outcomes in patients with a history of cardiovascular disease [198]. These differences may in part be due to genetic variations in the population [195] and warrants further investigation.

Future studies targeting neutrophils in chronic inflammation need to consider methods to limit the side effects of treatments. Neutrophils are critical in the acute inflammatory response to infection, and the complete ablation of neutrophils will lead to increased risks of infection, as with the antagonism of CXCR2 in COPD [173, 190] and delayed healing [199]. Possible methods to limit side effects are chronopharmacology and targeted drug delivery with nanoparticles. Recent findings highlight the importance of considering circadian rhythm in the treatment of inflammatory disease [200]. For example, time optimization of CCR2 neutralization results in limited macrovascular myeloid cell recruitment and atheroprogression without disturbing microvascular recruitment in Apoe-deficient mice fed a HFD [201]. Another method to optimize effects is through spacial targeting and can be performed by nanoparticle formulations that are directed to certain tissues. Collagen IV-targeted nanoparticles were used to selectively deliver the PAD4 inhibitor GSK484 to regions with endothelial cell shedding and collagen IV exposure in Apoe-deficient mice. Targeted treatment resulted in reduced NET accumulation and the attenuation of the risk for plaque rupture at sites of intimal injury [202]. In a model of IBD in mice, delivery of a proresolving annexin A1-mimetic peptide using oxidation-responsive nanoparticles that released their content at sites with high expression of ROS resulted in decreased colon inflammation and enhanced efferocytosis of apoptotic neutrophils [203]. Chronopharmacology and targeted delivery are promising new fields of study in the search for efficient and safe treatment strategies in chronic inflammatory disease.

## Conclusion

Neutrophils are commonly involved in several chronic inflammatory diseases and mainly exacerbate disease through different mechanisms, including neutrophil accumulation within the tissue, protease release and NET formation. Thus, several studies have focused on targeting neutrophil function as a possible treatment. In this sense, most strategies were centered on reducing neutrophil recruitment and inhibiting protease release and NET formation. As neutrophils contribute to many chronic diseases and have similar components, treatment strategies that work in one disease might be beneficial in other chronic diseases and warrant further investigation. Finally, future studies should aim to limit side effects such as increased infection propensity, which could be accomplished by chronopharmacology or targeted delivery of drugs.
